# *In Situ* Analyses Directly in Diarrheal Stool Reveal Large Variations in Bacterial Load and Active Toxin Expression of Enterotoxigenic *Escherichia* *coli* and *Vibrio cholerae*

**DOI:** 10.1128/mSphere.00517-17

**Published:** 2018-01-24

**Authors:** Yasmin Ara Begum, Hanna A. Rydberg, Kaisa Thorell, Young-Keun Kwak, Lei Sun, Enrique Joffré, Firdausi Qadri, Åsa Sjöling

**Affiliations:** a International Centre for Diarrhoeal Disease Research, Bangladesh, Centre for Health and Population Research, Dhaka, Bangladesh; b Department of Microbiology, Tumor and Cell Biology, Centre for Translational Microbiome Research (CTMR), Karolinska Institutet, Stockholm, Sweden; c Department of Microbiology and Immunology, Institute of Biomedicine, University of Gothenburg, Gothenburg, Sweden; U.S. Centers for Disease Control and Prevention

**Keywords:** ETEC, *Vibrio cholerae*, diarrhea, enterotoxin, quantification

## Abstract

The bacterial pathogens enterotoxigenic *Escherichia coli* (ETEC) and *Vibrio cholerae* are major causes of diarrhea. ETEC causes diarrhea by production of the heat-labile toxin (LT) and heat-stable toxins (STh and STp), while *V. cholerae* produces cholera toxin (CT). In this study, we determined the occurrence and bacterial doses of the two pathogens and their respective toxin expression levels directly in liquid diarrheal stools of patients in Dhaka, Bangladesh. By quantitative culture and real-time quantitative PCR (qPCR) detection of the toxin genes, the two pathogens were found to coexist in several of the patients, at concentrations between 10^2^ and 10^8^ bacterial gene copies per ml. Even in culture-negative samples, gene copy numbers of 10^2^ to 10^4^ of either ETEC or *V. cholerae* toxin genes were detected by qPCR. RNA was extracted directly from stool, and gene expression levels, quantified by reverse transcriptase qPCR (RT-qPCR), of the genes encoding CT, LT, STh, and STp showed expression of toxin genes. Toxin enzyme-linked immunosorbent assay (ELISA) confirmed active toxin secretion directly in the liquid diarrhea. Analysis of ETEC isolates by multiplex PCR, dot blot analysis, and genome sequencing suggested that there are genetic ETEC profiles that are more commonly found as dominating single pathogens and others that are coinfectants with lower bacterial loads. The ETEC genomes, including assembled genomes of dominating ETEC isolates expressing LT/STh/CS5/CS6 and LT/CS7, are provided. In addition, this study highlights an emerging important ETEC strain expressing LT/STp and the novel colonization factor CS27b. These findings have implications for investigations of pathogenesis as well as for vaccine development.

**IMPORTANCE** The cause of diarrheal disease is usually determined by screening for several microorganisms by various methods, and sole detection is used to assign the agent as the cause of disease. However, it has become increasingly clear that many infections are caused by coinfections with several pathogens and that the dose of the infecting pathogen is important. We quantified the absolute numbers of enterotoxigenic *E. coli* (ETEC) and *Vibrio cholerae* directly in diarrheal fluid. We noted several events where both pathogens were found but also a large dose dependency. In three samples, we found ETEC as the only pathogen sought for. These isolates belonged to globally distributed ETEC clones and were the dominating species in stool with active toxin expression. This suggests that certain superior virulent ETEC lineages are able to outcompete the gut microbiota and be the sole cause of disease and hence need to be specifically monitored.

## Abstract (precis)

The cause of diarrheal disease is usually determined by screening for several microorganisms by various methods, and sole detection is used to assign the agent as the cause of disease. However, it has become increasingly clear that many infections are caused by coinfections with several pathogens and that the dose of the infecting pathogen is important. We quantified the absolute numbers of enterotoxigenic *E. coli* (ETEC) and *Vibrio cholerae* directly in diarrheal fluid. We noted several events where both pathogens were found but also a large dose dependency. In three samples, we found ETEC as the only pathogen sought for. These isolates belonged to globally distributed ETEC clones and were the dominating species in stool with active toxin expression. This suggests that certain superior virulent ETEC lineages are able to outcompete the gut microbiota and be the sole cause of disease and hence need to be specifically monitored.

## INTRODUCTION

Diarrhea is the second leading cause of mortality in children younger than 5 years worldwide (1). Two of the major causes of severe diarrhea in low-resource countries, and for travelers to these countries, are the bacterial pathogens *Vibrio cholerae* (O1), which causes epidemic cholera, and enterotoxigenic *Escherichia coli* (ETEC) (2–4). These Gram-negative bacteria both infect the small intestine where they, by means of colonization factors (CFs), i.e., specific proteinaceous polymers, attach to the surface of the endothelial cells. An important CF for *V. cholerae* is the toxin-coregulated pilus (TCP) (5–7). For ETEC, more than 25 various CFs have to date been identified, of which the globally most prevalent ones are the CFA/I and the coli surface antigens 1 to 6 and 21 (CS1 to CS6 and CS21, respectively) (8–10). In some geographical areas, other coli surface antigens are also frequent, for example, CS7, CS14, and CS17 in Bangladesh (11). Both *V. cholerae* and ETEC are also defined by their toxin secretions. *V. cholerae* secretes cholera toxin (CT), whereas ETEC secretes heat-labile toxin (LT) and heat-stable toxin (ST). ST from ETEC strains pathogenic to humans consists of two variants, STh and STp (12, 13). The ETEC LT and the *V. cholerae* CT are in fact very similar in structure as well as function, and they cross-react immunologically (3, 14). These two toxins both bind to GM1 receptors on intestinal epithelial cells and trigger increased levels of intracellular cyclic AMP (cAMP), activation of protein kinase A (PKA), and subsequent activation of the ion channel cystic fibrosis transmembrane conductance regulator (CFTR). These steps of activation result in massive outflow of water and electrolytes from the epithelial cells with severe diarrhea as a result (3). The STs of ETEC cause diarrhea in a similar way, also deregulating the CFTR receptor, but by binding to guanylate cyclase receptors instead of GM1 receptors. For ETEC, individual isolates typically coexpress several CFs and/or toxins, such as CS5/CS6 with LT/STh and CS7 with LT. The genes encoding colonization factors and toxins are often located on the same plasmids (15, 16). Whole-genome sequencing has revealed that some ETEC strains carrying specific combinations of toxins and CFs on a conserved genomic background have spread globally, leading to the development of ETEC clades expressing certain CF-toxin combinations that are stable over time (10).

*V. cholerae* and ETEC are similar at symptom level, and in multipathogen diarrheas, ETEC is often underestimated or even missed (2). Coinfections with two or more diarrhea-causing pathogens are common. For example, it has been shown that for European travelers to the tropics, coinfections with various pathogens, including ETEC, enteroaggregative *E. coli* (EAEC), *Shigella*, and norovirus, were found in 61% of the diarrhea-suffering patients (17). With the development of new, molecular biology-based, diagnostic techniques, the probability of detecting all diarrhea-causing agents with high sensitivity increases (2). With these new techniques, underestimation of the role of ETEC in gastroenteritis outbreaks has been highlighted (18) and disease clinically diagnosed as cholera has been rediagnosed as ETEC after virulence determination using PCR (19). The incidence of coinfections by *V. cholerae* with ETEC is thus more common than previously thought. As an example, for patients attending the hospital ward at the International Centre for Diarrhoeal Diseases Research in Bangladesh (icddr,b), 2 to 16% of the patients were found positive for both *V. cholerae* and ETEC (11, 20). These findings prompted us to study the relationship between ETEC and *V. cholerae* in cases of severe diarrheal coinfections further. In particular, we aimed to study the CF/toxin profiles of the ETEC strains, both in single infection and in coinfection. Using qualitative culture, real-time quantitative PCR (qPCR) of the toxin profiles, and full-genome sequencing, directly in the diarrheal stool sample as well as in cultured isolated bacterial strains, new information about ETEC colonization factor/toxin profiles in coinfections with other *E. coli* strains and/or with *V. cholerae* could be retrieved. These pieces of information might be of importance for studies of ETEC virulence and pathology as well for the development of vaccines against ETEC.

## RESULTS

### Coinfections with ETEC, *V. cholerae*, and other enterobacteria are frequent in watery diarrhea.

During the diarrheal peak period in March to April 2006, 35 surveillance stool samples were randomly collected from children and adults seeking care for diarrheal disease at the hospital ward at icddr,b in Dhaka, Bangladesh. Seven collected samples did not meet the inclusion criteria and were therefore not analyzed. A total of 28 samples from children (*n* = 11, ages 2 to 17 years, median age of 12 years) and adults (*n* = 17, ages 18 to 54 years, median age of 35 years) were included in the study. The routine standard analyses for detection and surveillance of diarrheal pathogens at the icddr,b are culture analysis on MacConkey agar plates followed by multiplex PCR analysis on 3 to 10 pooled colonies for pathogenic *E. coli* and culture on selective taurocholate-tellurite-gelatin agar (TTGA) plates for detection of *V. cholerae*. Stool samples are by routine also tested for *Salmonella* spp. and *Shigella* spp. on selective plates. By use of these methods, 18 MacConkey agar plates were found positive for growth of *E. coli*-like bacteria, either as only *E. coli* (4 of the 18 plates) or as mixtures of *E. coli* with other bacteria, i.e., *Klebsiella* and other non-lactose fermenters, and/or *V. cholerae*, as presented in Table 1. Of the original 28 samples, 17 were positive for *V. cholerae*, which corresponds to 60%. All *V. cholerae*-positive samples were also found positive for other enterobacteria. None of the samples contained *Salmonella* or *Shigella*. Furthermore, the 18 samples positive for *E. coli* bacteria were tested for the presence of ETEC by multiplex PCR, showing that ETEC was present in 8 of the samples, corresponding to 29% of all the collected samples. Coinfections with ETEC and *V. cholerae* were found in 4 samples, corresponding to 14% of all the collected samples (Table 1). No significant correlation with age groups was found for either single infections or coinfections. Of the seven samples for which only *E. coli* colonies were detected on the MacConkey plates, four (samples 4, 5, 16, and 18) showed no growth of *V. cholerae*. These samples tested positive for ETEC toxins by use of multiplex PCR, suggesting that ETEC was the major pathogen.

**TABLE 1:** Diarrheal stool samples and presence of *E. coli*, ETEC, and *V. cholerae* by culture analysis

Stool sample/ collection no.	Date collected (mo-day)	Diarrhea type	MacConkey culture^a^	*V. cholerae* culture^b^	ETEC toxin + CF^c^	ETEC % of total *E. coli*^d^
1/001	3-20	Rice water	*E. coli* mix	Inaba	STh CF−	ND
2/002	3-20	Rice water	*E. coli* mix	Inaba		
3/005	3-21	Rice water	*E. coli* mix			
4/007	3-21	Brown watery	*E. coli* pure		LT CS7	100
5/008	3-22	Brown watery	*E. coli* pure		STh/LT CS5/CS6	100
6/009	3-22	Yellow watery	*E. coli* pure	Ogawa		
7/013	3-27	Yellow	*E. coli* pure	Ogawa		
8/014	3-27	Rice water	*E. coli* mix			
9/018	3-30	Yellow water	*E. coli* mix	Ogawa	STp CF−	60
10/020	3-30	Yellow water	*E. coli* mix	Inaba		
11/023	4-2	Yellow water	*E. coli* pure	Ogawa		
12/024	4-3	Yellow water	*E. coli* mix	Ogawa		
13/025	4-3	Yellow water	*E. coli* mix			
14/EN 94	4-3	Yellow water	*E. coli* mix	Ogawa		
15/026	4-4	Rice water	*E. coli* mix	Ogawa	LT CF−	0
16/027	4-4	Rice water	*E. coli* pure		STp/LT CF−	66 mix^e^
17/030	4-5	Rice water	*E. coli* mix	Ogawa	LT CF−	4
18/033	4-5	Brown rice water	*E. coli* pure		STp/LT CF−	96

^a^ Culture on MacConkey agar plates; mix, other nonfermenting and fermenting colonies detected indicating the presence of *Klebsiella* and other species; *E. coli* pure, only *E. coli*-like colonies detected.

^b^ Presence of *V. cholerae* detected by growth on TTGA plates; the serotypes Inaba and Ogawa were detected by agglutination tests.

^c^ Toxin and colonization factor profiles were determined on isolated colonies tested by multiplex PCR for toxin genes and CF dot blot analysis (48).

^d^ Percent ETEC per total *E. coli* bacteria was determined by toxin multiplex PCR performed on 50 individual *E. coli* colonies from MacConkey plates. (The number of positive colonies was divided by 50 tested *E. coli* colonies for each sample.) ND, not determined.

^e^ Sample 027 was a mix of several ETEC strains; of the 50 tested, 33 were ETEC, 4 were only LT postive, 2 were only STh positive, 20 were STp and LT positive, 1 was LT and STh positive, and 6 were positive for STh, STp, and LT.

### Determination of the ratio of ETEC CFU to total *E. coli* CFU in the diarrhea samples.

ETEC was detected in the samples where only *E. coli* colonies, and no other lactose-fermenting bacteria, grew on MacConkey agar. To determine the ETEC frequency in these samples, the numbers of ETEC CFU per total *E. coli* CFU were examined. The total numbers of *E. coli* in the samples were determined by quantitative culturing using serial dilutions. Isolated colonies, 50 in total, were picked randomly from the dilution plates and analyzed by toxin multiplex PCR. As presented in Table 1, the ETEC percentage of total number of *E. coli*-like CFU was then calculated by dividing the ETEC-positive colonies by 50, i.e., the total number of analyzed colonies. For two of the samples, sample 4 and sample 5, 50 out of 50 colonies were determined as positive for either LT (sample 4) or both LT and STh (sample 5), suggesting that they were pure ETEC. For sample 18, 96% of the colonies were determined as ETEC expressing LT and STp. For sample 9, 60% of the *E. coli* strains were STp positive, while in sample 17, only 4% were LT positive. The results from the individual colonies largely agreed with initial toxin profiles determined for the diarrheal samples, with the exception of sample 16. This sample was originally scored as an *E. coli* LT/STp-only infection, but in the analysis of the 50 colonies, it was found to contain multiple ETEC toxin profiles (Table 1). In addition, the fraction of ETEC in *E*. *coli* in the sample was 66%, indicating a mixed infection with several ETEC strains and other *E. coli* strains. Regarding sample 1, this sample was not tested, and in sample 15, the original ETEC isolate could not be found among the tested 50 colonies. In total, six representative isolates, E2264 to E2269, were collected from the remaining six ETEC-positive samples and stored in freeze medium. The colonization factor profiles of these strains were then tested using dot blot and PCR analysis, showing the presence of CS7 in sample 4 and CS5/CS6 in sample 5. For the other ETEC strains, the CF profiles could not be determined (Table 1).

### qPCR quantification of ETEC and *V. cholerae* toxin genes.

Since variations in ETEC frequency occur in diarrheal stool, we next sought to determine the absolute numbers of ETEC and *V. cholerae* per milliliter of watery stool. The DNA copy numbers of ETEC and *V. cholerae* were determined in the 18 *E. coli*-positive stool samples by use of qPCR analysis and primers specific for *estA1* STp, *estA2* to *estA4* STh, *eltB* LT, and *ctxB* CT together with standard curves of known copy numbers. Gene loci for all four toxin genes were detected in a majority of the samples, and the amounts varied between 0 copies and 2 × 10^8^ copies per ml, as presented in Table 2. Higher numbers of toxin gene copies were found in samples that tested positive for ETEC toxins than in samples that were negative for ETEC in the previous culture analyses. Samples 4, 5, 16, and 18, which were all positive for LT ETEC, contained between 1 × 10^7^ and 6 × 10^7^ LT gene copies per ml. Furthermore, sample 4 and sample 5 contained, in comparison with the others, very low copy numbers (0 and 150 copies per ml stool, respectively) of the *V. cholerae* CT gene. Hence, these samples likely represent true ETEC-only diarrheas. The two other LT ETEC-positive samples, sample 15 and sample 17, contained few or no LT ETEC bacteria per total amount of *E. coli* in the culture analysis, and the levels of ETEC LT gene copies detected by qPCR were correspondingly 3 orders of magnitude lower (2 × 10^4^ to 3 × 10^4^) than in the other ETEC-positive samples. The copy number of *eltB* was additionally found at levels between 10^3^ and 10^5^ copies per ml in the samples that tested negative for ETEC in culture.

**TABLE 2:** Absolute gene copy numbers for *ctxB* (CT), *eltB* (LT), *estA2* to *estA4* (STh), and *estA1* (STp) calculated by qPCR on DNA extracted directly from 1 ml of liquid diarrhea^a^

Sample (presence of ETEC and/or *V. cholerae* toxins)	Copy no./ml				CFU/ml	
	*ctxB* CT	*eltB* (LT)	*estA2* to *estA4* (STh)	*estA1* (STp)	Total *E. coli*	ETEC (% of total *E. coli*)
1/001 (STh/**CT**)	**7.79 × 10^6^**	8.6 × 10^4^	**2.97 × 10^6^**	0	ND	ND
2/002 (**CT**)	**3.88 × 10^7^**	5.9 × 10^4^	8.5 × 10^3^	0	ND	
3/005 (–)	1.29 × 10^4^	1.97 × 10^5^	1.31 × 10^4^	0	3.0 × 10^5^	
4/007 (**LT**)	0	**3.75 × 10^7^**	3.6 × 10^3^	0	6.7 × 10^8^	6.7 × 10^8^ (100)
5/008 (**LT** + STh)	1.51 × 10^2^	**1.09 × 10^7^**	**2.12 × 10^7^**	0	2.7 × 10^7^	2.7 × 10^7^ (100)
6/009 (**CT**)	**1.1 × 10^4^**	5.29 × 10^4^	4.4 × 10^3^	0	ND	
7/013 (**CT**)	**8.91 × 10^5^**	1.75 × 10^3^	4.0 × 10^2^	0	5.5 × 10^7^	
8/014 (–)	7.6 × 10^1^	1.52 × 10^4^	3.1 × 10^3^	0	7.8 × 10^6^	
9/018 (STp/**CT**)	**1.55 × 10^6^**	1.10 × 10^4^	4.65 × 10^3^	**4.3 × 10^3^**	1.0 × 10^7^	6.0 × 10^6^ (60)
10/ 020 (**few CT**)	3.74 × 10^2^	5.95 × 10^3^	1.07 × 10^4^	9.0 × 10^2^	2.5 × 10^7^	
11/023 (**CT**)	**4.59 × 10^4^**	1.14 × 10^4^	3.8 × 10^3^	4.85 × 10^3^	8.5 × 10^7^	
12/024 (**CT**)	**6.68 × 10^5^**	1.5 × 10^4^	3.45 × 10^3^	8.2 × 10^3^	8.0 × 10^5^	
13/025 (–)	3.42 × 10^2^	6.0 × 10^3^	1.3 × 10^3^	3.35 × 10^3^	8.0 × 10^6^	
14/EN 94 (**CT**)	**5.46 × 10^4^**	4.4 × 10^3^	3.0 × 10^3^	3.95 × 10^3^	2.0 × 10^5^	
15/026 (**LT**/**CT**)	**1.74 × 10^8^**	**2.78 × 10^4^**	8.55 × 10^3^	2.04 × 10^4^	2.5 × 10^7^	0
16/027 (**LT + STh + STp**)	3.62 × 10^2^	**4.25 × 10^7^**	**7.15 × 10^7^**	**2.5 × 10^4^**	7.0 × 10^6^	4.62 × 10^6^ (66)
17/030 (**LT**/**CT**)	**1.2 × 10^5^**	**1.78 × 10^4^**	2.05 × 10^3^	5.55 × 10^3^	9.6 × 10^4^	3.84 × 10^3^ (4)
18/033 (**LT + STp**)	5.23 × 10^5^	**6.25 × 10^7^**	9.5 × 10^2^	**1.83 × 10^6^**	6.3 × 10^7^	6.05 × 10^7^ (96)

^a^ Copy numbers corresponding to samples with culture-positive *V. cholerae* and/or ETEC and corresponding toxin profiles are indicated in bold. Quantitative culture of total amount of *E. coli* in the liquid diarrhea samples was used to estimate total number of *E. coli* bacteria in samples. The percentage of ETEC CFU determined on multiplex PCR analysis of 50 individual colonies was used to calculate the assumed numbers of ETEC CFU per milliliter of diarrhea. ND, not determined.

The gene encoding heat-stable toxin STp was detected in the STp-positive sample 9 (9 × 10^3^ copies) and sample 16 (2 × 10^4^) and in high numbers in sample 18 (2 × 10^8^), as presented in Table 2. Sample 18 was by quantitative culture and multiplex PCR determined to contain 6.3 × 10^7^ *E. coli* bacteria per ml diarrheal fluid, and 96% of the colonies were LT and STp positive in culture analysis. Hence, the qPCR results and quantitative culture corroborate a dissemination concentration of 10^7^ to 10^8^ gene equivalents per ml diarrheal stool in this patient. A similar concentration was also found for sample 5, which contained 100% ETEC and a total *E. coli* count of 2.7 × 10^7^ CFU per ml. The LT and STh gene copy numbers of this sample were 1 × 10^7^ (*eltB*) and 2 × 10^7^ (*estA3* and *estA4*), respectively. The gene copy numbers for STh were for sample 16 estimated to be 7 × 10^7^ and for sample 1 estimated to be 3 × 10^7^, whereas all samples that were negative for STh in culture analyses showed levels of between 10^2^ and 10^4^ copies of *estA2* to *estA4* per ml (Table 2).

The gene counts for *V. cholerae ctxB* in the analyzed samples varied from none to almost 2 × 10^8^ gene copies per ml (Table 2). Low (≤200 copies) or absent levels were found in sample 4 and sample 5, both detected as 100% ETEC. Low levels (≤400 copies) were also found in samples 8, 10, 13, and 16. For sample 10, only around 25 colonies in total of *V. cholerae* were detected on culture plates, which corroborates the low counts detected with qPCR. In the other samples with low copy numbers of *ctxB*, samples 8, 13, and 16, no growth of *V. cholerae* was consequently detected in culture analysis. The samples that scored positive for *V. cholerae* in culture showed gene copy numbers of between 10^4^ and 10^8^ per ml. In addition, sample 18, which was detected as *V. cholerae* negative by culture, also contained 5 × 10^5^ *ctxB* copies per ml. Taken together, these results suggest that low levels of ETEC and *V. cholerae* are continuously present in a majority of diarrheal stools from patients and that these levels are difficult to detect by routine culture analyses.

### Toxin production and secretion determined directly in diarrheal liquid stool samples by GM1-ELISA.

The diarrheal stool samples were further analyzed for the presence of the translated ST, LT, and CT by use of GM1 enzyme-linked immunosorbent assay (GM1-ELISA). Seven of the eight stool samples that had been scored as positive for ETEC were tested using GM1-ELISA and inhibition GM1-ELISA (Table 3). In the three tested samples that were culture positive for *V. cholerae* (samples 1, 9, and 15), CT was detected by LT-39, an antibody that detects both LT and CT, as well as CT-Wi monoclonal antibody (MAb), which is more specific for CT. Cholera toxin was also detected in sample 18, which was estimated to contain approximately 5 × 10^5^ *V. cholerae* bacteria per ml by qPCR analysis but was *V. cholerae* negative in culture. The toxin ELISA results thus confirmed the qPCR results for this sample. Two of the samples, sample 4 and sample 5, were detected as virtually pure ETEC samples, with undetectable levels of *V. cholerae* toxin in qPCR. For sample 4, traces of LT were detected in the pellet fraction using the LT-specific MAb LT-80, but the amount was close to the lower detection limit of the assay. In sample 5, however, positive results were obtained using MAbs LT-80 and LT-39 but not MAb CT-Wi, suggesting that the toxin found indeed was LT and not CT. In addition, this sample was positive only in the bacterial supernatant fraction and not in the pellet, indicating that LT was actively secreted during infection. ST was detected in the supernatants of sample 1, sample 9, and sample 16, for which STh or STp had already been detected by toxin multiplex PCR. Sample 5, additionally, showed trace amounts of ST. Furthermore, ST was detected in sample 15, in both pellet and supernatant, whereas the same toxin was not detected by culture analysis followed by multiplex PCR, and only approximately 10^4^ copies of *estA2* to *estA4* were detected by real-time PCR analysis (Table 2). In contrast, no ST was detected in sample 18, for which high levels of STp had been detected by both culture/multiplex PCR and real-time PCR analysis. The analyses show that toxins are present in diarrheal stool and distributed to the environment. In addition, both ETEC and *V. cholerae* evidently actively secrete LT and CT during acute infection and dissemination.

**TABLE 3:** ETEC and cholera toxins detected in ETEC-positive diarrhea samples from patients^a^

Stool sample	Identified ETEC strain	Cholera toxin	ETEC toxin	Amt of toxin (ng/ml)							
				LT-39		LT-80		CT-Wi		ST-1	
				Pellet	Supernatant	Pellet	Supernatant	Pellet	Supernatant	Pellet	Supernatant
1	NR	CT	STh		15.8						61.3
4	E2264		LT	Trace		Trace					
5	E2265		STh/LT		25.2		24				Trace
9	E2266	CT	STp	4.8	4.8			2.2	3.4		53
15	NR	CT	LT	24.2	24.2			22	40.3	26	15
16	E2267		STp/LT								20.3
17	E2268	CT	LT	ND	ND	ND	ND	ND	ND	ND	ND
18	E2269		STp/LT	4.9	24.2			4.9	16.8		

^a^ Production and secretion of the toxins were quantified by GM1-ELISA. The LT-39 MAb detects both CT and LT, while the other MAbs are specific for ST (both STh and STp), LT, and CT, respectively. The pellet fraction represents toxins associated with the bacterial fraction, and the supernatant represents the amount of secreted toxin in the sample. NR, not recovered; ND, not determined.

### Gene expression of the ETEC and *V. cholerae* toxin genes.

Toxin levels in stool might not indicate active transcription and translation. To investigate whether active toxin transcription occurs in ETEC and *V. cholerae* in diarrheal stool, the expression of the toxin genes in the two pathogens was measured by extracting RNA from the bacterial pellet from liquid diarrheal samples and reverse transcription of the RNA to cDNA. The relative expression of the *ctxB* (CTB subunit) gene, the ETEC toxin genes *estA2* to *estA4* and *estA1* (STh and STp, respectively), and *eltB* (LTB subunit) was determined in a fixed concentration of total RNA converted to cDNA (15 ng/PCR mixture). The mRNA expression of the CT-, LT-, and ST-encoding genes per 15 ng total cDNA was found to largely correspond to the toxin profiles, as seen in Table 4. Higher gene expression levels of the *ctxB* mRNA were found in samples 1, 9, and 12 and particularly in sample 15 compared to the other samples. These samples also had correspondingly high levels (2 × 10^5^ to 2 × 10^8^) of *ctxB* DNA copies (Table 2). The highest mRNA levels for *eltB* and the ST-encoding mRNAs were found in samples 1, 4, 5, 16, and 18, all confirmed to have large amounts of ETEC (Tables 1 and 2). For sample 15, no gene expression of ETEC mRNA toxin genes could be determined, whereas sample 8, for which no pathogen was found in culture, showed expression of *eltB* and *estA2* to *estA4*. These results, together with the DNA data, suggest that an LT and STh-positive ETEC infection was missed in culture analysis in this patient. The results show that toxin gene expression levels generally correlate with numbers of the respective pathogen in the stool. Calculations of expressed gene copy numbers (mRNA) divided by genomic copy numbers (DNA), i.e., gene expression per genome equivalent, showed that toxin gene expression levels in ETEC and *V. cholerae* are similar.

**TABLE 4:** Gene copy numbers determined by RT-qPCR^c^

Stool sample	ETEC strain	Cholera toxin	ETEC toxin	Copy no./15 ng of cDNA for gene:			
				*ctxB*	*eltB*	*estA2* to *estA4*	*estA1*
1/001	x^a^	**CT**	**STh**	**118**	5.6	**92**	
3/005				55			
4/007	E2264		**LT**		**2,705**		
5/008	E2265		**STh/LT**		**823**	**80,223**	
8/014					24	151	
9/018	E2266	**CT**	STp	**44**			
12/024		**CT**		**167**	0.2		
15/026	x^a^	**CT**	**LT**	**1,880**			
16/027	E2267		**STp/LT**^b^		**227**	**3,969**	
17/030	E2268	**CT**	**LT**	**0.07**	**0.07**	1.3	
18/033	E2269		**STp/LT**	3.4	**4,020**		**25,887**

^a^ Isolate not recovered.

^b^ This sample was a mixture of several different ETEC isolates.

^c^ Correlation between toxin profile determined by multiplex PCR and detection of corresponding gene is indicated in bold.

### Whole-genome sequencing of ETEC isolates.

The results presented above suggest that ETEC might be underestimated in cases of cholera-like liquid diarrhea, as well as that certain ETEC clones such as LT CS7 (sample 4) and LT STh CS5 plus CS6 (sample 5) can manifest as monocultures of a single clonal infection. In order to investigate the genetics of the collected ETEC strains and to be able to correlate the collected ETEC with worldwide ETEC infections, whole-genome sequence (WGS) analysis was performed. For isolates E2264 (sample 4) and E2265 (sample 5) PacBio sequencing was performed to gain even more complete information about the genetic details. These two strains were of specific interest since they were detected in ETEC-only infections and since they belong to ETEC lineages that have persisted over time and have spread globally (10). The four other recovered isolates, E2266 (sample 9), E2267 (sample 16), E2268 (sample 17), and E2269 (sample 18), were sequenced using Illumina MiSeq sequencing. The details of the sequencing are provided in Tables S1 to S4 in the supplemental material. The genomes of the six isolates were annotated and analyzed using CGE *in silico* multilocus sequence typing (MLST), PlasmidFinder, and plasmid MLST (pMLST) as well as ResFinder VirulenceFinder and ARG-annot. In addition, pathogenic *E. coli* virulence genes were identified using BLAST analysis.

10.1128/mSphere.00517-17.1 TABLE S1  Assembly and annotation statistics of the PacBio sequencing of E2264 and E2265. Download TABLE S1, DOCX file, 0.02 MB. Copyright © 2018 Begum et al. 2018 Begum et al. This content is distributed under the terms of the Creative Commons Attribution 4.0 International license.

10.1128/mSphere.00517-17.2 TABLE S2  Assembly and annotation statistics of the Illumina sequencing of E2266 to E2269. Download TABLE S2, DOCX file, 0.02 MB. Copyright © 2018 Begum et al. 2018 Begum et al. This content is distributed under the terms of the Creative Commons Attribution 4.0 International license.

10.1128/mSphere.00517-17.3 TABLE S3  ETEC virulence factors identified in the ETEC strains. Download TABLE S3, DOCX file, 0.02 MB. Copyright © 2018 Begum et al. 2018 Begum et al. This content is distributed under the terms of the Creative Commons Attribution 4.0 International license.

10.1128/mSphere.00517-17.4 TABLE S4  Antibiotic resistance genes found in the ETEC strains. Download TABLE S4, DOCX file, 0.02 MB. Copyright © 2018 Begum et al. 2018 Begum et al. This content is distributed under the terms of the Creative Commons Attribution 4.0 International license.

*In silico* MLST confirmed that E2265 (sample 5) belongs to the major ETEC lineage 5 (ST-443) and that E2264 belongs to the novel ST-5305, a sublineage within ETEC lineage 3 (10). The STs identified for E2266 (sample 9), E2267 (sample 16), E2268 (sample 17), and E2269 (sample 18) were ST-226, ST-100, ST-5474, and ST-4493, respectively. A search in the Achtman MLST database (https://enterobase.warwick.ac.uk/warwick_mlst_legacy) revealed that ST-226 seemingly is associated with enteroaggregative *E. coli* (EAEC) as well as with CS26-positive ETEC (21). ST-100 (E2267, sample 16) has previously been found in porcine ETEC and also recently in human ETEC isolates (22). ST-5474 and ST-4493 have, in contrast, not previously been associated with ETEC. ST-4493 has been found in environmental *E. coli* isolates, whereas ST-5474 was not recorded in the Achtman database.

The genomes were furthermore searched for CF and other virulence genes, as well as for antibiotic resistance. The E2264 (sample 4) isolate was determined as LT/CS7 ETEC and also showed predicted antibiotic resistance to sulfonamide (*sul2*), beta-lactams (*ampC* and *bla*_TEM-1b_), tetracycline (*tetB*), trimethoprim (*dfrA8*), and streptomycin (*strAB*). The E2269 (sample 18) isolate was shown to harbor the LT, STp, and CS27b virulence genes. The last is a member of the CS18/CS18-like family of emerging new putative ETEC colonization factors (21, 22). E2269, additionally, harbored putative resistance genes for sulfonamide (*sul2*), beta-lactams (*ampC* and *bla*_TEM-1b_), tetracycline (*tetA*-like), trimethoprim (*dfrA1*), erythromycin [*mph(A)*-like], and streptomycin (*strAB* and *aadA1*). For isolate E2265, which was detected as an LT/STh/CS5/CS6 ETEC, the CF profile could be verified, and for isolate E2268, a CS13/CS23-like colonization factor could be detected. For both E2265 and E2268, no predicted antibiotic resistance genes were found using ResFinder, while *ampC* was detected by ARG-annot, indicating chromosomally encoded ampicillin resistance. E2266, originally scored as an STp ETEC, was predicted to be resistant to beta-lactams (*ampC* and *bla*_TEM-1b_), trimethoprim (*dfrA1*), and erythromycin [*mph(A)*-like]. The WGS data analysis could not, however, confirm any virulence genes for this isolate. Moreover, E2267, scored as LT STh, was predicted to be resistant to beta-lactams (*ampC*), streptomycin (*aadA1*), and trimethoprim (*dfrA1*), and the colonization factor was determined as CS14. Furthermore, all strains, except E2268, contained the plasmid segregation protein ParM, which has been identified as ETEC specific (23).

### PacBio analysis of E2264 and E2265 revealed large virulence plasmids and additional plasmids with transfer systems and antibiotic resistance.

Since two stool samples, sample 4 and sample 5, contained 100% ETEC of two commonly isolated lineages of ETEC, PacBio sequencing was employed to further analyze these isolates. The PacBio analysis revealed that ETEC 2264 (sample 4), in addition to the chromosome, contained 3 plasmids. The largest plasmid (E2264_p112045) had a size of 112,045 bp and contained 128 putative open reading frames (ORFs). Among these, the ORFs containing the *tra* genes *traD* and *traI*, which may have helicase activity, as well as the genes encoding the heat-labile enterotoxin A and B subunits for production of LT (orf66 and orf67) and the CS7 operon subunit precursor (orf75 to orf81), were found. The distance between LT and CS7 was 3,986 bp, and the sequence contained two conserved, hypothetical proteins and four transposase genes. The six CS7 operon genes found were, in 5′ to 3′ direction, positioned in the order D, F, E, C, B, and A. The second largest plasmid (E2264_p77345) of ETEC 2264 was 77,345 bp long and contained 93 ORFs. This plasmid contained a high number of *tra* genes and antibiotic resistance genes. The *tra* genes, which are involved in conjugative transfer of plasmids between bacteria, encountered were *traA*, *traD*, *traE*, *traG*, *traH*, *traK*, *traL*, *traM*, *traN*, *traP*, *traQ*, *traT*, *traU*, *traV*, *traX*, and *traY*. Among these genes are the genes encoding the pilus subunit (*traA*), genes for regulation of *traA*, and genes for pilus assembly as well as genes for nicking and unwinding of DNA (24–26). In addition, six ORFs involved in antibiotic resistance were found: ones for resistance to tetracycline (orf63), trimethoprim (orf81), beta-lactams (orf83), and sulfonamide (orf87) and finally *strA* (orf88) and *strB* (orf89), coding for streptomycin resistance. The third plasmid (E2264_p45777) of ETEC 2264 was 45,777 bp long and contained 45 ORFs. This plasmid contained the gene encoding the secreted autotransporter serine protease EatA (ETEC autotransporter A) (orf25) (27). Sequence comparison of EatA using BLAST (NCBI) demonstrated high homology with a vast number of sequences of *E. coli* origin (97 to 99% homology with 7 samples, ≥71% homology with 84 samples) and also with a few sequences from *Shigella*.

ETEC 2265 contained two plasmids in addition to the chromosome. The largest plasmid, E2265_p142359, was 142,359 bp long and harbored 189 ORFs, including genes encoding important virulence factors like *eatA* (orf66), the *csfA* to *-F* operon encoding CS5 (orf79 to orf84), the *cssABCD* operon encoding CS6 (orf96 to orf99), and the *estA3* and *estA4* gene encoding STh (orf107). The plasmid also contained an *aatPABCD* operon encoding a membrane transporter initially described in enteroaggregative *E. coli* (EAEC) and a *cexA*-like gene located directly upstream of *aatPABC* (28). Plasmid E2265_p142359 also contained several *tra* genes: *traM*, *traJ*, *traA*, *traL traE*, *traY*, *traK*, *traB*, and *traP*. The second plasmid, E2265_p88757, was 88,757 bp long and contained 101 ORFs, including the *eltAB* operon (orf81 and orf82), as well as a high number of *tra* genes: *traM*, *traA*, *traL*, *traE*, *traK*, *traB*, *traV*, *traC*, *trbL*, *traW*, *traU*, *trbC*, *traN*, *traF*, *trbB*, *traH*, *traG*, *traS*, *traT*, *traD*, *traI*, and *traX*.

### Scoary analysis of the genomes did not reveal any unique single-pathogen infection profiles for the ETEC.

**FIG 1:**
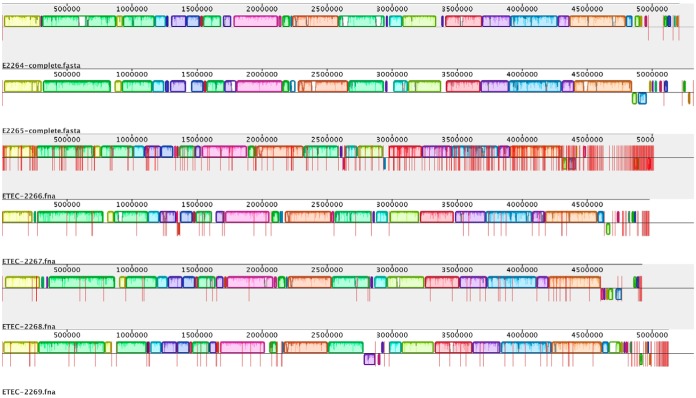
Mauve analysis of the six ETEC isolates E2264, E2265, E2266, E2267, E2268, and E2269. ETEC isolates E2264 and E2265 were PacBio sequenced and contain one chromosome and three and two plasmids, respectively, that are separated by lines in the figures. The other four ETEC isolates were sequenced by Illumina sequencing with individual contigs separated by lines in the figure. Genome analysis revealed conserved chromosomal organization, as represented by colors in the figure. The total pan-genome contained 7,537 genes, and the core genome consisted of 3,502 genes.

The results indicate that three of the diarrheal samples collected in this study, E2264, E2265, and E2269, might be true ETEC infections without other coinfecting pathogens. In order to investigate whether these isolates differed from the three isolates with ETEC found as mixed infections with other *E. coli* strains and/or with *V. cholerae* (E2266, E2267, and E2268), the pan-, core, and accessory genomes were determined for the six sequenced isolates (Fig. 1). The pan-genome of the six isolates comprised 7,537 genes; of these, 3,502 genes were common to all isolates and constituted the core genome. Scoary analysis to determine if genes were significantly associated with the three ETEC-only infections did not reveal any significant results. The only differences found were larger genomes of E2264, E2265, and E2269 than of isolates E2266, E2267, and E2268 (see Table S1 in the supplemental material).

## DISCUSSION

In this study, the prevalence and virulence factor profiles of ETEC and *V. cholerae* in watery stool from patients with cholera-like diarrhea were investigated by use of genetic sequencing and molecular analysis as well as traditional culture methods. In total, 28 samples were randomly collected at the hospital ward at icddr,b in Dhaka, Bangladesh. Of these, 17 tested positive for *V. cholerae* and 8 tested positive for ETEC (Table 1). Quantitative culture of bacterial load on MacConkey plates and analysis of ETEC toxin profile by PCR found that some samples were dominated by ETEC while others were mixed with other bacteria. Three of the ETEC samples, samples 4, 5, and 18, were detected as pure or almost pure ETEC infections, while samples 1, 9, 15, 16, and 17 were found as coinfectants with *V. cholerae* and/or with various undetermined *E. coli* and *Enterobacteriaceae* strains. Coinfections with various pathogens are quite common among patients with diarrhea (29, 30). For example, Paschke et al. have previously reported a prevalence of coinfections in as many as 60.5% of travelers to tropical and subtropical parts of the world who suffer from diarrhea as well as in 12.5% of the travelers to the same areas with no disease symptoms (17). The coinfection rate of the two pathogens investigated in this work was found to be 14%, which is higher than the 2% previously reported by Chowdhury et al. (11) but similar to the results from the work of Begum et al. (20) in the same geographical area.

Since both *V. cholerae* and ETEC cause massive diarrheas with a daily loss of several liters of fluid during the acute phase, we wanted to investigate the concentration of the bacteria in the stool samples. By use of quantitative real-time PCR, we could determine the gene copy number of the genes encoding the disease-causing *V. cholerae* and ETEC toxins, to up to 10^8^ copies per ml (Table 2). These numbers were corroborated by the results of the quantitative culture and other studies reporting between 10^7^ and 10^8^ CFU of diarrheal pathogens per milliliter or gram of stool (31, 32) and up to 10^8^ to 10^9^ pathogen gene copies per gram of stool (30). Toxin gene copy numbers in ETEC have been determined to be between 1 and 16 copies per cell (32, 33), indicating that quantification of gene copies might overestimate bacterial load up to 10-fold. Regardless, shedding of 10^7^ to 10^8^ bacteria per ml of stool in patients, who can lose several liters of fluid per day, is probably one of the main factors contributing to the large epidemic outbursts of diarrhea caused by these pathogens.

One of the findings of this work was that the use of highly sensitive molecular methods detects low levels of ETEC and *V. cholerae* in a majority of the stool samples negative in culture (Table 2). These low levels are probably not causing symptoms and may constitute a background pathogenic microbiota in individuals living in areas of endemicity. They could also mean that the rates of coinfections are significantly higher than previously reported. Recent studies have, however, highlighted the importance of pathogen load for disease manifestation (30, 34, 35). High levels of ETEC in stool were linked to clinical manifestation of diarrhea in ETEC-challenged volunteers (35). In the same study, it was found that *E. coli* 16S (i.e., ETEC) dominated the microbiota in volunteers who developed diarrheal symptoms but was low in asymptomatic volunteers. Even though severe diarrheas caused by ETEC and by *V. cholerae* are very similarly manifested, *V. cholerae* is frequently identified as the disease-causing pathogen. The development of quantitative molecular diagnostics, including real-time PCR and other PCR methods for identification of the pathogens, may aid in finding the major disease-causing pathogen(s) in an infection (30, 36). With the use of these techniques, ETEC has been shown to be frequently underestimated (32). It might also turn out that a high proportion of the cholera cases are, in fact, mixed infections, since we detected *E. coli* or other species in all *V. cholerae*-positive stool samples.

Although the heat-labile CT and LT of *V. cholerae* and ETEC have similar actions, cholera is generally regarded to cause a more severe disease outcome. This has been explained by differences in the amount of toxin that the bacteria secrete. *V. cholerae* is generally considered to secrete most of its produced CT, while ETEC retains >50% of the LT intracellularly, either in the periplasm or associated with the membrane lipopolysaccharides (LPS) under laboratory conditions (37, 38). ST is also considered to cause a more severe disease outcome than LT (39, 40), and infection with LT ETEC has subsequently often been detected in asymptomatic patients (17). The reason for this difference is unknown but might be addressed by the difference in secretion. Thus, in order to study the toxin secretion in more detail, the 8 samples that by culture analysis were identified to contain ETEC were further analyzed for both ETEC and *V. cholerae* toxin production and secretion (Table 3). Using quantitative ELISA and monoclonal antibodies specific for the different toxins, it was seen that for *V. cholerae*, CT was detected both in the bacterial fraction and in the supernatant, indicating that approximately one-third of the toxin produced was actually retained in the bacteria (Table 3). For ETEC, similar levels of LT were detected both in the bacterial cells and in the supernatant, suggesting that LT is secreted to a relatively large extent. ST was mainly found as secreted toxin, but for one of the samples, ST was localized to the bacterial cells. This implies that the concept that *V. cholerae* always secretes CT and that ST is always secreted by ETEC, while most LT is retained in the periplasm of ETEC, does not hold true during infection. Our results indicate that disseminating ETEC and *V. cholerae* actively both transcribe and secrete toxins during shedding in diarrheal stool, which further supports the epidemic nature of these pathogens. Since ETEC toxins were also found secreted in the liquid diarrhea in levels that were equal to *V. cholerae* and since the toxin gene expression levels and pathogen loads were similar for the two pathogens, the explanation for the more severe nature of *V. cholerae* infections is still elusive. However, since these results are from only a few stool samples, further studies are needed.

Quantitative culture of bacterial load and analysis of ETEC toxin profile by PCR in this study found that some samples were dominated by ETEC while others were mixed with other bacteria. Six ETEC isolates were recovered and used for further analysis. Half of the ETEC isolates, E2264, E2265, and E2269, from samples 4, 5, and 18, respectively, were detected as pure or almost pure ETEC infections. The other isolates, E2266, E2267, and E2268, recovered from samples 9, 16, and 17, respectively, were found as coinfectants with *V. cholerae* and with various undetermined *E. coli* and *Enterobacteriaceae* strains (Table 1). All the 6 isolated strains were whole genome sequenced, using Illumina or Pacific Bioscience (PacBio) sequencing, in order to further analyze the genomic content.

Moreover, the toxin and colonization factor profiles of the isolated ETEC strains were investigated. For E2264 (sample 4) and E2265 (sample 5), the genetic analysis could confirm the CF profiles detected by dot blot analysis of CS7 and CS5/CS6, as well as toxin profiles of LT and LT/STh, respectively (Table 1). These toxin/CF profiles are common and widespread over the world (10). The E2264 isolate belongs to a subtype of ETEC lineage 3 expressing LT and CS7, which is frequently isolated globally (10, 20, 41). The E2265 isolate belonged to ETEC lineage 5, a clonal group that expresses the toxins LT and STh and colonization factors CS5 and CS6. Recent reports indicate that ETEC expressing LT/STh/CS5/CS6 is the most common ETEC pathotype isolated in Dhaka (20). Both these strains were detected as pure ETEC infections in this study, suggesting that these toxin/CF profiles are beneficial for single infections. A study performed in Dhaka in 2011, using whole-genome sequencing of several isolates recovered from individual patients in a setup similar to this study, identified one patient for whom all analyzed isolates constituted a clonal expansion of L5 (LT/STh CS5/CS6) isolates (42), which further confirms that L5 isolates are able to outcompete the normal microbiota and cause serious infections. The other four strains were detected as CF negative in the initial dot blot analysis; however, sequence analysis revealed CF profiles for three of them: E2267 (sample 16) was detected as CS14 positive, E2269 (sample 18) was detected positive for CS27b, and E2268 (sample 17) was shown to harbor genes for a CF profile similar to CS13/CS23. No CF could be detected for E2266 (sample 9), suggesting that this strain either lacks CF or expresses a kind of colonization factor that has not yet been discovered or that the plasmid(s) was lost prior to sequencing. Nonetheless, this STp/CF combination rendered a strain that was the major infecting pathogen of the *E. coli* species still beneficial for coinfections. The strain E2267 showed an STp/LT/CS14 profile, whereas E2268 was shown positive for LT in combination with new CFs that are very similar, but not identical, to CS13/CS23. Both ETEC strains were found in coinfections. The E2269 strain also showed a new toxin-CF combination. This novel type of ETEC, expressing LT, STp, and CS27b, is worth keeping an eye on. It was detected in an almost pure ETEC infection and is definitely potent as a single-infection pathogen. The LT/STp/CS27b ETEC of MLST ST-4493 was not described during the time of isolation of the strains in this paper. The novel CF CS27b was first described by Nada and coworkers in 2011 (21). Recent publications have indicated that ETEC strains previously noted as CF negative might express a novel group of CS18- and CS20-like CFs, to which CS27b belongs (21, 22). These new CFs are not detectable using traditional dot blot techniques and might be missed using the present PCR methods in use; therefore, more information about the various ETEC toxin-CF combinations is needed. The matter is, however, complex, and no consensus regarding the CF/toxin profiles and disease outcome has been determined to date (9).

Next, we sought to determine why certain ETEC toxin-CF combinations manifest as single infections. Scoary analysis of the genomes, however, did not reveal any unique gene profiles that could explain the single-pathogen infections seen for samples 4, 5, and 18. Analysis of antibiotic resistance genes was performed to determine if single infections are more resistant. Two of the single-pathogen infectants, E2264 (sample 4) and E2269 (sample 18), showed multiple genes for antibiotic resistance. In contrast, using ResFinder, no acquired resistance genes were found for E2265 (sample 5), while ARG-annot confirmed chromosome-bound ampicillin resistance by *ampC* (see Table S4 in the supplemental material). This isolate has been described previously (43, 44). Regarding the ETEC strains found as coinfecting pathogens, E2266 (sample 9) and E2267 (sample 16) harbored four and three antibiotic resistance genes each, respectively. Also in this group, one of the three strains, this time E2268 (sample 17), harbored no presumed plasmid-borne resistance genes except *ampC*. For E2268, the lack of acquired resistance genes coincides with the lack of the ETEC-specific ParM gene (23), which was detected in all the other strains. This strain might thus be somewhat less potent, considering that only 4% of the *E. coli* isolates of this sample were determined as ETEC, or it might be highly specialized in occurring in coinfections. The high frequency of resistance genes detected in some isolates might not be surprising considering that a test of the drinking water in Dhaka some years ago revealed that 36% of the *E. coli* isolates were multiresistant, of which 26% were positive for extended-spectrum beta-lactamases (45). The presence of antibiotic resistance might thus not be important for diarrheal virulence.

Nevertheless, in this work we have shown, although with a limited number of samples, that there might be specific CF/toxin profiles associated with either single ETEC infections or multipathogen infections. Two of the isolates, E2264 (sample 4) and E2265 (sample 5), were found to belong to globally successful ETEC lineages that have been described previously (10). Hence, although the isolates described in this work were collected a decade ago, they are indeed still relevant and frequently detected pathogens. Here, we used second- (Illumina) and third-generation (PacBio) sequencing technologies, the latest developed in the last 5 years, which allowed us to perform *de novo* assemblies and characterize ETEC plasmids. The genome data will be useful for deeper studies of pathogen genomics.

Recent studies on global diarrhea in the GEMS and MAL-ED studies have identified STh-expressing ETEC to be a major contributor to diarrhea (40). The identification in this study of potent single infections by LT/CS7 and LT/STp/CS27b ETEC does indicate that focus on STh-expressing ETEC might be an oversimplification. Indeed, Del Canto et al. recently described clonal CS27b ETEC isolated from Chile, Pakistan, India, and Bangladesh (22), indicating that LT/STp/CS27b is an emerging virulent ETEC type. Given this, we propose that preventive efforts and vaccine strategies against ETEC should be focused on globally spread ETEC lineages.

## MATERIALS AND METHODS

### Ethics statement.

The collected samples were part of the icddr,b 2% surveillance system routine, approved by the Research Review Committee (RRC) and Ethical Review Committee (ERC) of icddr,b, Dhaka, Bangladesh, as described previously (10, 20). Exclusion criteria in this study were the presence of agents other than *Enterobacteriaceae* and *Vibrio* or if the patient reported having had antibiotic treatment prior to hospitalization. Informed oral consent was obtained from adult patients, or from caregivers or guardians of children, for collection of stool specimens, according to the hospital policy. The ERC has approved verbal consent and voluntary participation, and subjects may refuse participation without compromise of patient care. All patients were treated for their clinical conditions, e.g., dehydration, after sample collection. Consenting individuals were assured about the nondisclosure of name or identity of the participants. The ETEC strains collected and analyzed in this study were deposited at the ETEC culture collection of the University of Gothenburg and in the group of Å. Sjöling. Permission to use the ETEC strain collection was granted by the Regional Ethical Board of Gothenburg, Sweden (Ethics Committee reference 088-10).

### Bacterial growth and detection of ETEC and *V. cholerae.*

Watery diarrheal stool samples were collected from the hospital ward at the International Centre for Diarrhoeal Diseases Research in Bangladesh (icddr,b) during the diarrheal peak season in March to April 2006 and brought to the adjacent laboratory for culture of bacteria. The clinical criteria for admission were moderate to severe watery diarrhea requiring hospitalization.

The collected liquid diarrheal samples were serially diluted in phosphate-buffered saline (PBS) and cultured on MacConkey agar plates for determination of CFU of *E. coli* per milliliter. *E. coli* was distinguished from other lactose-fermenting bacteria, including *Klebsiella*, *Citrobacter*, and *Enterobacter*, by ocular investigation. Only the *E. coli*-like colonies were picked for subsequent analyses. The same serial diluted samples were cultured in parallel on selective taurocholate-tellurite-gelatin agar (TTGA) plates to determine growth of *V. cholerae* (46). The *V. cholerae* serotype, Inaba or Ogawa, was identified by an agglutination test (47). To verify the presence of ETEC among the samples containing *E. coli*-like bacteria, ETEC toxin multiplex PCR was performed as described previously (48). In short, 6 to 10 colonies from the MacConkey agar plates were pooled, boiled in 500 µl MilliQ water, and tested for the presence of the genes encoding the ETEC toxins LT, STh, and STp. Individual colonies were collected from the ETEC-positive plates, and the toxin profile was retested and confirmed by multiplex PCR. One representative isolate was saved in freeze medium at −80°C. Furthermore, the colonization factor profiles of these representative isolates were determined by multiplex PCR and by dot blot analysis, as previously described (48, 49).

### Determination of the proportion of ETEC to total number of *E. coli* bacteria in stool.

To determine the percentage of ETEC per total number of *E. coli-*like colonies in the diarrheal stool samples, 50 *E. coli*-like colonies were randomly collected from the original ETEC-positive MacConkey agar plates. The colonies were individually boiled for 10 min in MilliQ water and tested by toxin multiplex PCR (48). The percentage of ETEC per total *E. coli* bacteria in each sample was calculated by dividing the number of toxin-positive colonies by 50 (total number of analyzed *E. coli* colonies).

### Collection of bacterial pellet and supernatant from diarrheal liquid for DNA and RNA analysis.

From the original watery diarrheal samples, DNA and RNA were extracted for molecular quantification of DNA as well as for gene expression analyses of both ETEC and *V. cholerae*. The diarrheal samples were first centrifuged at 1,000 × *g* for 5 min to separate mucus and solid particles from the bacterium-containing supernatant. From the remaining supernatant, three separate samples of 1 ml each were collected and additionally centrifuged for 5 min at 16,000 × *g* followed by subsequent separation of bacterial pellet and supernatant. The first centrifuged sample was saved for subsequent determination of the amounts of toxins secreted by the bacteria or associated with the bacterial cells by GM1-ELISA (described below). For this sample, the pellet was dissolved in PBS and sonicated before it was frozen at −70°C, and the corresponding supernatant was frozen separately at −70°C. These samples were stored at maximum for 1 day at −70°C, after which they were analyzed. For the second and third 1-ml samples from the same stool sample, the bacterial pellets were collected and immediately frozen at −70°C for subsequent DNA and RNA extraction, respectively.

### Detection of ETEC and cholera toxins in diarrhea samples by GM1-ELISA.

In order to detect the toxins produced by ETEC and *V. cholerae* in the diarrheal samples, the pellet and supernatant samples were analyzed by GM1-ELISA for detection of CT and LT and by inhibition GM1-ELISA for detection of ST. The procedures have been described previously (48, 50). With the use of in-house monoclonal antibodies specific for ST (ST-1), LT (LT-80), and CT (CT-Wi), as well as an antibody that recognizes both LT and CT (LT-39), the total amount of ST, LT, or CT could be determined in the supernatant (secreted toxin) and in the pellet fraction (toxin associated with the bacterial cell membrane or cytoplasm). All antibodies were produced at the department of Microbiology and Immunology, Sahlgrenska Academy, University of Gothenburg. Threefold dilution series and reference toxin standards of known concentrations were used to determine the amount of each toxin present in both pellet and supernatant of the samples.

### DNA and RNA extraction from diarrhea samples.

The second and third 1-ml samples from the diarrhea sample preparation were used for DNA and RNA extraction and subsequent qPCR analyses of gene copy numbers and gene expression. Bacterial DNA extractions from the frozen pellets were performed with the QIAamp stool DNA kit (Qiagen, Hilden, Germany) as described by the manufacturer and in previous studies (33, 51). The extracted DNA was kept at −20°C until analysis. RNA extraction was performed with the RNeasy kit (Qiagen, Hilden, Germany), and a DNase protocol (Qiagen) was included to remove genomic DNA as described previously (52). Extracted RNA was analyzed on an agarose gel to determine integrity, and the concentration was measured at 260 nm using a NanoDrop spectrophotometer (NanoDrop Technologies, Wilmington, DE, USA). cDNA was prepared from 200 ng RNA from each sample using the QuantiTect cDNA kit (Qiagen) with an additional DNase step included in the protocol. The cDNA was stored at −20°C until further analysis.

### qPCR quantification of ETEC and *V. cholerae* in diarrhea samples.

Quantitative PCR (qPCR) was performed to determine the total amount of ETEC bacteria in the DNA samples using primers specific for the *eltB* (LT), *estA1* (STp), and *estA2* to *estA4* (STh) genes (33) and *ctxB* (CT) (52). For ETEC, a standard curve for each gene was generated by PCR amplification using the respective real-time PCR primer and a toxin-positive ETEC strain as the DNA template. The PCR products were purified using the QIAquick PCR purification kit (Qiagen), and the concentrations were determined on a NanoDrop spectrophotometer (NanoDrop Technologies, Wilmington, DE, USA). The PCR product copy number was determined as described previously using Avogadro’s number and the molecular weight of the PCR product (53). Tenfold serial dilutions between 5 × 10^8^ and 5 copies/µliter were prepared and stored at −20°C until further use. To determine the total number of *V. cholerae* bacteria in the diarrheal sample, real-time PCR was performed using the same conditions as for ETEC. A standard curve was generated by manually counting the bacteria of the N16961 El Tor *V. cholerae* reference strain using a Neubauer improved counting chamber (Hausser Scientific, VWR International) at a magnification of ×40. Counted bacteria were diluted in 10-fold serial dilutions to the same concentrations as described above and used as a standard curve. Real-time PCRs were run in duplicates in 96-well plates (Applied Biosystems) with a total volume of 20 μl in each reaction mixture. The PCR mix contained 10 μl SYBR green real-time PCR master mix (Life Technologies), 10 pmol of each primer, 6 μl water, and 2 μl DNA. Negative controls and a standard curve were included in each PCR run. The numbers of bacteria per 1 ml liquid diarrheal sample were calculated by using the settings for absolute quantification in the ABI 7500 real-time PCR instrument and by assuming that one gene copy equals one bacterium.

### Determination of toxin gene expression per bacterium in stool samples.

The same primers and qPCR conditions were used in reverse transcriptase qPCR (RT-qPCR) for gene expression analysis. As the template, the cDNA was used, and each sample was analyzed in duplicate. A no-RT reaction mixture containing only RNA for each sample was run in duplicate to confirm that the detected expression was not due to genomic DNA amplification. The real-time PCR was run on an ABI 7500 using SYBR green and standard amplification conditions, as described above, in a reaction volume of 20 µl. The gene copy number per bacterial genome was calculated by dividing the numbers of gene transcripts per milliliter of sample with the gene copy number per milliliter of sample as described previously (52).

### Whole-genome Illumina sequencing.

In order to investigate the genetics of the collected ETEC strains, and to be able to correlate the collected ETEC with worldwide ETEC infections, whole-genome sequence (WGS) analysis was performed. For E2264 (sample 4) and E2265 (sample 5), PacBio sequencing was performed to gain even more complete information about the genetic details since these strains were of specific interest due to their being found in ETEC-only infections (described below). The four other ETEC strains were sequenced using Illumina MiSeq sequencing. The ETEC strains, stored at −80°C, were plated on LB agar plates and incubated for 24 h at 37°C. One large bacterial colony from each plate was selected and washed in 300 µl MilliQ water, after which the bacterial DNA was extracted using the DNeasy Blood & Tissue kit from Qiagen according to the manufacturer’s instructions. The DNA concentration was measured using a Qubit 2.0 fluorometer (Invitrogen). Sequencing libraries were prepared using the TruSeq Nano kit (Illumina, San Diego, CA) with a mean fragment length of 900 bp. Libraries were sequenced on the MiSeq platform v.3 chemistry, 2 by 300 bp, generating a coverage of >100× for all strains.

### PacBio sequencing.

DNA for Pacific Biosciences (PacBio) sequencing was prepared from ETEC isolates grown in LB medium to an optical density at 600 nm (OD_600_) of 0.3. DNA was extracted by the Qiagen Genomic-tip 500/G kit according to the manufacturer’s instructions (Qiagen, Hilden, Germany). For each sample, one DNA aliquot was sheared into 10-kbp fragments using a Genemachines HydroShear instrument (Digilab, Marlborough, MA, USA) and a second aliquot was sheared into 2-kb fragments using a Covaris instrument (Covaris, Woburn, MA). SMRTbell templates were constructed according to the manufacturer’s instructions (Pacific Biosciences, Menlo Park, CA, USA). Each library was sequenced on 1 SMRT cell on a Pacific Biosciences RSII sequencer according to the manufacturer’s instructions with 4-h movie time.

### Assembly and annotation.

The reads from the 10-kb PacBio sequencing library were assembled using HGAP3 from SMRTportal v2.3 (Pacific Biosciences, Menlo Park, CA, USA) with default settings. The 2-kb PacBio libraries were assembled using Falcon (Pacific Biosciences, Menlo Park, CA, USA) with settings allowing high coverage for plasmid assembly.

Illumina raw reads were trimmed and filtered using TrimGalore! (54), applying the quality cutoff Q30 and keeping only reads longer than 30 bp. Filtered reads were *de novo* assembled using SPAdes v 3.10.1 (55), and resulting assembly files were filtered for very-low-coverage contigs and contigs shorter than 500 bp before they were ordered to the E2265 complete PacBio sequence using the Mauve order contigs tool (56).

The resulting draft and complete genomes were annotated with the *prokka* annotation pipeline v. 1.1.12b (57) using the E24377A (CP000800.1) ETEC proteome as primary annotation source. Summary statistics from the sequencing, assembly, and annotation were collected using MultiQC v1.0 (58) and are shown in Table S1 (PacBio) and Table S2 (Illumina) in the supplemental material.

### Functional and comparative genomic analysis.

To perform initial functional analysis, we used the CGE pipeline v 1.1 (59), which performs resistance gene prediction using ResFinder, *in silico* MLST, plasmid prediction, and pMLST. Resistance gene prediction was also performed by ARG-annot (http://en.mediterranee-infection.com/article.php?laref=283%26titre=arg-annot).

Whole-genome alignment was performed and visualized using progressiveMauve v. 2015/2/25 (60). Pan-genome analysis was performed using Roary v. 3.6.2 (61), using a blastp identity cutoff of 85%. Comparison between the ETEC-only infection genomes and mixed-infection genomes was performed using the Scoary tool v. 1.6.10 (62).

### Statistical analyses.

Statistical analyses were performed using GraphPad Prism version 7.0. *P* values of <0.05 were considered significant.

### Accession number(s).

The sequences of E2266 to E2269 have been deposited at DDBJ/ENA/GenBank under the accession numbers NQYN00000000 (E2266), NQYM00000000 (E2267), NQYL00000000 (E2268), and NQYK00000000 (E2269). The versions described in this paper are versions NQYN01000000, NQYM01000000, NQYL01000000, and NQYK01000000, respectively. The assembled chromosome and three plasmids of E2264 have been deposited in GenBank under accession numbers CP023349, CP023350, CP023351, and CP023352, respectively. The assembled chromosome and two plasmids of E2265 have been deposited in GenBank under accession numbers CP023346, CP023347, and CP023348, respectively.
