# Supplementary material for: In Situ Analyses Directly in Diarrheal Stool Reveal Large Variations in Bacterial Load and Active Toxin Expression of Enterotoxigenic Escherichia coli and Vibrio cholerae
Source: mSphere. 2018 Jan 24;3(1):e00517-17. doi: 10.1128/mSphere.00517-17 (PMC5784243; doi:10.1128/mSphere.00517-17)
Supplement: TABLE S1 [file sph001182460st1.docx]

Table S1

| **Sample** | **Contigs** | **Bases** | **Coding sequences** | **Signal Peptides** | **rRNA** | **tRNA** | **tmRNA** | **Repeat regions** |
| --- | --- | --- | --- | --- | --- | --- | --- | --- |
| E2264-chr | 1 | 4972828 | 4681 | 428 | 22 | 94 | 1 | 4 |
| E2264-p1 | 1 | 112045 | 128 | 10 |  |  |  |  |
| E2264-p2 | 1 | 77345 | 93 | 12 |  |  |  |  |
| E2264-p3 | 1 | 45777 | 45 | 1 |  |  |  |  |
| E2265-chr | 1 | 5088339 | 4802 | 434 | 22 | 94 | 1 | 2 |
| E2265-p1 | 1 | 142368 | 189 | 11 |  |  |  |  |
| E2265-p2 | 1 | 88757 | 101 | 12 |  |  |  |  |
