# Supplementary material for: In Situ Analyses Directly in Diarrheal Stool Reveal Large Variations in Bacterial Load and Active Toxin Expression of Enterotoxigenic Escherichia coli and Vibrio cholerae
Source: mSphere. 2018 Jan 24;3(1):e00517-17. doi: 10.1128/mSphere.00517-17 (PMC5784243; doi:10.1128/mSphere.00517-17)
Supplement: TABLE S2 [file sph001182460st2.docx]

Table S2

| **Sample** | **Contigs** | **Bases** | **Coding sequences** | **Signal Peptides** | **rRNA** | **tRNA** | **tmRNA** | **Repeat regions** | **Fold Seq. coverage** |
| --- | --- | --- | --- | --- | --- | --- | --- | --- | --- |
| ETEC-2266 | 638 | 5010060 | 4727 | 389 | 6 | 88 | 1 | 3 | 167 |
| ETEC-2267 | 86 | 4977694 | 4721 | 433 | 8 | 88 | 1 | 2 | 164 |
| ETEC-2268 | 57 | 4920382 | 4612 | 445 | 8 | 86 | 1 | 2 | 100 |
| ETEC-2269 | 112 | 5125183 | 4805 | 462 | 9 | 83 | 1 | 2 | 176 |
