# Supplementary material for: In Situ Analyses Directly in Diarrheal Stool Reveal Large Variations in Bacterial Load and Active Toxin Expression of Enterotoxigenic Escherichia coli and Vibrio cholerae
Source: mSphere. 2018 Jan 24;3(1):e00517-17. doi: 10.1128/mSphere.00517-17 (PMC5784243; doi:10.1128/mSphere.00517-17)
Supplement: TABLE S3 [file sph001182460st3.docx]

Table S3

| **Strain** | **MLST 1** | **Toxin** | **CF** | **plasmids** | **IncF** | **pMLST summary** |
| --- | --- | --- | --- | --- | --- | --- |
| E2264 | ST-5305 | LT | CS7 | IncI1,IncFII(pCoo) | F12*:A-:B- | IncI1[Unknown ST],IncF[F12*:A-:B-] |
| E2265 | ST-443 | LT, ST A3/A4 | CS5/CS6 | IncFII,IncFII(pCoo) | F11:A-:B- | IncF[F11:A-:B-] |
| E2266 | ST-226 | - | - | IncI1,Col(BS512),Col8282,  ColRNAI |  | IncI1[Unknown ST] |
| E2267 | ST-100 | LT, ST A3/A4 | CS14 | IncFII(29),IncFIB(AP001918),ColRNAI | F30:A-:B24 | IncF[F30:A-:B24] |
| E2268 | ST-5474 | - | CS23/CS13-like | IncFII,IncFIB(AP001918) | F19*:A-:B55* | IncF[F19*:A-:B55*] |
| E2269 | ST-4493 | LT, ST-IA/ST-Psta1 | CS27b | IncFII(29),IncFIB(AP001918),Col(MGD2),Col8282,Col156,ColRNAI | F30:A-:B59* | IncF[F30:A-:B59*] |
