# Supplementary material for: In Situ Analyses Directly in Diarrheal Stool Reveal Large Variations in Bacterial Load and Active Toxin Expression of Enterotoxigenic Escherichia coli and Vibrio cholerae
Source: mSphere. 2018 Jan 24;3(1):e00517-17. doi: 10.1128/mSphere.00517-17 (PMC5784243; doi:10.1128/mSphere.00517-17)
Supplement: TABLE S4 [file sph001182460st4.docx]

Table S4

| **Strain** | **Genome size** | **Contigs** | **Aminoglycoside** | **Sulphonamide** | **Beta-lactam** | **Tetracycline** | **Trimethoprim** | **Macrolide** |
| --- | --- | --- | --- | --- | --- | --- | --- | --- |
| E2264 | 5207995 | 4 | strA,strB-like | sul2 | ampC blaTEM-1B | tet(B) | dfrA8 |  |
| E2265 | 5319464 | 3 |  |  | ampC |  |  |  |
| E2266 | 5010060 | 638 |  |  | ampC blaTEM-1B |  | dfrA1 | mph(A)-like |
| E2267 | 4977694 | 86 | aadA1 |  | ampC |  | dfrA1 |  |
| E2268 | 4920382 | 57 |  |  | ampC |  |  |  |
| E2269 | 5125183 | 112 | aadA1,strA,strB | sul2 | ampC blaTEM-1B | tet(A)-like | dfrA1 | mph(A)-like |
